# Organotypic Cultures of Intervertebral Disc Cells: Responses to Growth Factors and Signaling Pathways Involved

**DOI:** 10.1155/2015/427138

**Published:** 2015-10-25

**Authors:** Harris Pratsinis, Dimitris Kletsas

**Affiliations:** Laboratory of Cell Proliferation & Ageing, Institute of Biosciences & Applications, National Centre for Scientific Research “Demokritos”, 153 10 Athens, Greece

## Abstract

Intervertebral disc (IVD) degeneration is strongly associated with low back pain, a major cause of disability worldwide. An in-depth understanding of IVD cell physiology is required for the design of novel regenerative therapies. Accordingly, aim of this work was the study of IVD cell responses to mitogenic growth factors in a three-dimensional (3D) organotypic milieu, comprising characteristic molecules of IVD's extracellular matrix. In particular, annulus fibrosus (AF) cells were cultured inside collagen type-I gels, while nucleus pulposus (NP) cells in chondroitin sulfate A (CSA) supplemented collagen gels, and the effects of Platelet-Derived Growth Factor (PDGF), basic Fibroblast Growth Factor (bFGF), and Insulin-Like Growth Factor-I (IGF-I) were assessed. All three growth factors stimulated DNA synthesis in both AF and NP 3D cell cultures, with potencies similar to those observed previously in monolayers. CSA supplementation inhibited basal DNA synthesis rates, without affecting the response to growth factors. ERK and Akt were found to be phosphorylated following growth factor stimulation. Blockade of these two signaling pathways using pharmacologic inhibitors significantly, though not completely, inhibited growth factor-induced DNA synthesis. The proposed culture systems may prove useful for further in vitro studies aiming at future interventions for IVD regeneration.

## 1. Introduction

Low back pain has been reported to be the leading cause of disability worldwide [1] having a great impact on the health care system and society [2]. It is strongly associated with intervertebral disc (IVD) degeneration [3]. IVDs lie between the vertebral bodies of the spinal column providing mechanical support and flexibility to the body and absorbing the loads and vibrations that result from the standing position and the specific activities of each person [3]. IVDs consist of an outer layer of laminated fibres (containing fibroblast-like cells) and a gelatinous core (with cells resembling chondrocytes) called annulus fibrosus (AF) and nucleus pulposus (NP), respectively [3]. AF is characterized by a well-organized network of concentric collagen lamellae, with collagen type-I being the predominant extracellular matrix (ECM) constituent [4]. On the other hand, NP mostly comprises collagen type-II and proteoglycans, especially aggrecan, which maintains tissue hydration due to its chondroitin and keratan sulfate chains [3].

IVD degeneration is characterized by tissue disorganization and vascular and neural infiltration, a fact associated with the discogenic back pain [5]. Changes at the molecular and biochemical levels have been observed in the degenerated IVDs, such as loss of proteoglycans and water [6] and increased expression of matrix metalloproteinases and aggrecanase [7]. Many of these changes have been associated with alterations in the expression levels of various growth factors and their receptors [8, 9]. Currently, IVD degeneration is mainly treated with medication aiming at pain relief or—in more severe cases—with surgical interventions, such as discectomy, spinal fusion, or disc replacement, all of which however exhibit many clinical contraindications and possible catastrophic complications [10]. Hence, novel therapies aiming at the regeneration of the degenerated disc have been suggested, such as cell transplantation [11, 12] or growth factor injections [13, 14]. Nevertheless, for the successful outcome of such efforts, the in-depth understanding of disc cell physiology is necessary, especially regarding the proliferative responses to growth factors. We have previously reported that Platelet-Derived Growth Factor (PDGF), basic Fibroblast Growth Factor (bFGF), and Insulin-Like Growth Factor-I (IGF-I) stimulate the proliferation of bovine IVD cells in vitro via the activation of the ERK and Akt signaling pathways [15]. Furthermore, we have shown that the same growth factors added in human IVD cells, as well as autocrine factors produced by them, stimulate their proliferation via the same two signaling pathways [16]. These previous studies have been conducted using the conventional monolayer cell culture approach, which does not approximate very well the in vivo environment of the tissue. Accordingly, aim of the present report was the examination of bovine IVD cell proliferative responses to these three growth factors using three-dimensional (3D) culture systems. In an effort to simulate the cells' in vivo environment, proteins encountered in abundance in the two IVD compartments were used; that is, AF cells were cultured inside collagen type-I gels, while NP cells were cultured in collagen gels supplemented with chondroitin sulfate A (CSA).

## 2. Materials and Methods

### 2.1. Materials

Human recombinant (h.r.) PDGF-BB (the PDGF-isoform considered to represent the universal ligand for all PDGF receptor subtypes [17]), h.r. bFGF, and h.r. IGF-I were purchased from R&D Systems (Minneapolis, MN, USA). Chondroitin sulfate A sodium salt from bovine trachea (CSA), PD98059, wortmannin, LY294002, calphostin C, Y-27632 dihydrochloride, protease and phosphatase inhibitor cocktails, 5-bromo-2′-deoxyuridine (BrdU), 4′,6-diamidino-2-phenylindole dihydrochloride (DAPI), and goat anti-mouse and anti-rabbit horseradish peroxidase-conjugated secondary antibodies were obtained from Sigma (St. Louis, MO, USA). The rabbit anti-phospho-Akt (Ser473) and anti-Akt1/2/3 antibodies were obtained from Cell Signaling Technology (Hertfordshire, UK), while mouse anti-phospho-ERK1/2 antibody that recognizes phosphorylated Thr202/Tyr204 and mouse anti-pan-ERK antibodies were obtained from BD Transduction Laboratories (Bedford, MA, USA). The FITC-conjugated anti-BrdU monoclonal antibody (clone BMC9318) was from Roche Diagnostics GmbH (Mannheim, Germany). [Methyl-^3^H]-thymidine was from Moravek Biochemicals (Brea, CA, USA). Crude collagenase, cell culture media, antibiotics, and sodium pyruvate were purchased from Biochrom KG (Berlin, Germany), except for the low glucose (1,000 mg/L) formulation of Dulbecco's minimal essential medium (DMEM), trypsin, and fetal bovine serum (FBS) which were from Gibco, Life Technologies Europe BV (Thessaloniki, Greece).

### 2.2. Cell Isolation and Cell Culture Conditions

Tails from young steers (8–12 months of age) were obtained from a local slaughterhouse and they were processed within 8 h after slaughter, as described [15]. Briefly, nucleus pulposus (NP) and outer annulus fibrosus (AF) were isolated based on visual inspection, and each part was further minced in small pieces (approximately 1 mm^3^), which were subjected to an overnight digestion with a crude collagenase solution in DMEM (1 mg/mL for NP and 3 mg/mL for AF). Cells were recovered by centrifugation and they were routinely cultured in DMEM (high glucose formulation, i.e., 4,000 mg/L) supplemented with penicillin and streptomycin, sodium pyruvate, L-glutamine, and 10% FBS in a humidified atmosphere of 5% CO_2_ at 37°C. Cells were routinely subcultured when confluent by using a trypsin/citrate (0.25/0.30% w/v) solution. Cell counting, after trypsinization, was performed by using a Coulter counter (Beckman-Coulter, Fullerton, CA). Cells were tested periodically and found to be mycoplasma-free.

### 2.3. Preparation of Three-Dimensional Cell Cultures

Collagen was extracted from rat-tail tendons according to a modification of the method of Bell et al. [18]. Briefly, tendons were solubilized under aseptic conditions in 0.1% (v/v) acetic acid, for 48 h, at 4°C. The solution was centrifuged at 10,000 rpm in a Sorvall (DuPont) model RC-5C centrifuge in an SS-34 rotor for 90 min and the supernatant was stored at 4°C. This stock solution contained approximately 4 mg/mL total protein and consisted primarily of collagen type-I. Alternatively, a commercially available rat-tail collagen type-I solution was used (BD Biosciences, Bedford, MA, USA) containing 4.01 mg/mL total protein in 0.02 N acetic acid. Equivalent results were obtained using both collagen type-I solutions.

In order to form 3D cell-populated collagen gels, a premix of the collagen solution with DMEM 10x and NaHCO_3_ 7.5% at a ratio of 17 : 2 : 1 was prepared. This was used for AF cells or it was enriched with CSA at the indicated concentrations to be used for NP cells. Cell pellets (10^6^ cells/mL of solution) and FBS (at a final concentration of 0.1%) were added to the premix, mixed further to ensure a homogeneous distribution of the cells and layered on the culture dishes. After 30 min at 37°C for polymerization, low glucose formulation of DMEM containing 0.1% FBS was layered on top of the gels.

### 2.4. Tritiated Thymidine Incorporation Assay

3D cell cultures were left for 2 days in low glucose formulation of DMEM containing 0.1% FBS. Then fresh medium was added along with the growth factors to be tested and methyl-[^3^H]-thymidine (0.2 *μ*Ci/mL, 25 Ci/mmol). After 24 h of incubation, the medium was aspirated and the collagen gels were digested for 1 h at 37°C, with a crude collagenase solution (1 mg/mL in 130 mM NaCl, 10 mM CaCl_2_, 10 mM HEPES, pH 7.2). The cells were collected by centrifugation (1000 ×g, 5 min) and lysed in 0.3 N NaOH/1% SDS solution for 1 h. Ice-cold TCA (f.c. 10%) was added to the lysates, which were kept at 4°C for 1 h more. The lysates were then filtered through GF/B glass-fiber filters (Sigma). The filters were air-dried and subjected to scintillation counting, as previously described [19].

When indicated, the cells were preincubated with the appropriate concentrations of kinase inhibitors for 45 min before growth factor treatment.

### 2.5. Bromodeoxyuridine Incorporation Assay

IVD cells were plated overnight on glass coverslips at a density of 2 × 10^4^ cells/cm^2^, in DMEM containing 10% FBS. They were growth-arrested for 48 h in low glucose formulation of DMEM containing 0.1% FBS and then stimulated for 24 h with PDGF-BB (10 ng/mL) in medium supplemented with 50 *μ*M BrdU, in the presence or absence of CSA (250 *μ*g/mL). The cells were fixed in freshly prepared solution of 4% paraformaldehyde in PBS, permeabilized with 0.2% Triton X-100 in PBS, treated with 2 N HCl, and incubated with FITC-conjugated anti-BrdU-mAb (at 4°C, overnight) followed by staining with 1 mg/mL DAPI in PBS (10 minutes) in the dark at room temperature. Cells were washed 3 times with PBS at each step. DAPI- and BrdU-positive nuclei were observed on a Zeiss Axioplan 2 fluorescence microscope with a 40x objective; a field containing approximately 200 cells was used for quantification purposes. Images were captured using a ProgRes CF cool CCD camera (Jenoptik Optical Systems GmbH, Jena, Germany) controlled by a PC equipped with the ProgRes CapturePro software (Jenoptik).

### 2.6. Western Analysis

3D cell cultures were left for 2 days in low glucose formulation of DMEM containing 0.1% FBS. Then fresh medium was added along with the growth factors to be tested for the indicated time periods. For the collection of cell lysates, 3D gels were washed with ice-cold Tris buffered saline (TBS: 10 mM Tris-HCl, pH 7.4, 15 mM NaCl), carefully detached from the culture dishes and transferred to Eppendorf tubes. After a brief centrifugation (10,000 ×g, 3 min, 4°C), the supernatant was discarded, and gels including the cells were compacted to pellets. Hot SDS-PAGE sample buffer, that is, 62.5 mM Tris, pH 6.8, 6% w/v SDS, 2% v/v glycerol, 5% v/v 2-mercaptoethanol, 0.0125% w/v bromophenol blue, and protease and phosphatase inhibitor cocktails (Sigma), was added to the pellets, and following sonication for 15 s, the samples were clarified by centrifugation and stored at −80°C until use. The lysates were separated on SDS-PAGE (gradient 5%–12.5%) and the proteins were transferred to Polyscreen PVDF membranes (Perkin Elmer, Thessaloniki, Greece). The membranes were blocked with 5% (w/v) nonfat dried milk in 10 mM Tris-HCl, pH 7.4, 150 mM NaCl, and 0.05% Tween-20 (TTBS) buffer and incubated with the appropriate primary antibodies. After washing with TTBS, the membranes were incubated with either anti-mouse or anti-rabbit horseradish peroxidase-conjugated goat secondary antibody (Sigma) and washed again with TTBS and the immunoreactive bands were visualized by chemiluminescence (LumiSensor HRP Substrate Kit, GenScript, Piscataway, NJ, USA) according to the manufacturer's instructions on a Fujifilm LAS-4000 luminescent image analyzer (Fujifilm Manufacturing, Greenwood, SC, USA).

### 2.7. Statistical Analysis

Results were expressed as mean values ± SEM. Differences in the presented values were evaluated by Student's *t*-test.

## 3. Results

### 3.1. DNA Synthesis Stimulation by Growth Factors

**Figure 1:**
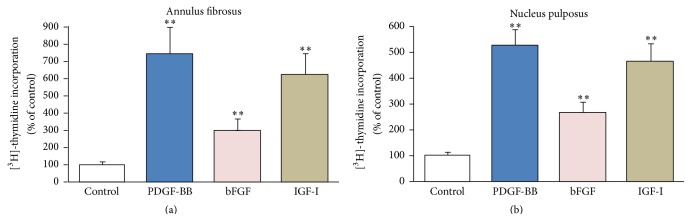
Effect of growth factors on DNA synthesis. Bovine AF (a) and NP (b) cells were cultured in collagen gels and collagen gels supplemented with CSA, respectively, and stimulated with PDGF (10 ng/mL), bFGF (5 ng/mL), and IGF-I (100 ng/mL) along with tritiated thymidine for 24 hours. Thymidine incorporation was assessed as described in Section 2. Values represent mean (±SEM) of three independent experiments. Asterisks (*∗∗*) indicate statistically significant differences compared to control (*p* < 0.01).

Primary bovine coccygeal AF cells cultured in 3D collagen type-I gels in the presence of a minute FBS concentration (0.1%) were arrested in the G_0_/G_1_ phase of the cell cycle as determined by flow cytometry after propidium iodide staining (not shown here). Under these conditions the cells exhibited intense DNA synthesis responses to PDGF-BB, bFGF, and IGF-I (Figure 1(a)). The highest response was elicited by PDGF (746%), followed by IGF-I (620%), and bFGF (301%). The responses to PDGF and IGF-I, however, did not differ statistically significantly, and only the response to bFGF was significantly lower compared to the other two growth factors. Similarly, bovine NP cells cultured in 3D collagen gels supplemented with CSA were also stimulated by the three growth factors with the same order of intensity (Figure 1(b)). In general, AF cell responses to each growth factor compared to the control were higher than the ones of NP cells; these differences however were not statistically significant.

**Figure 2:**
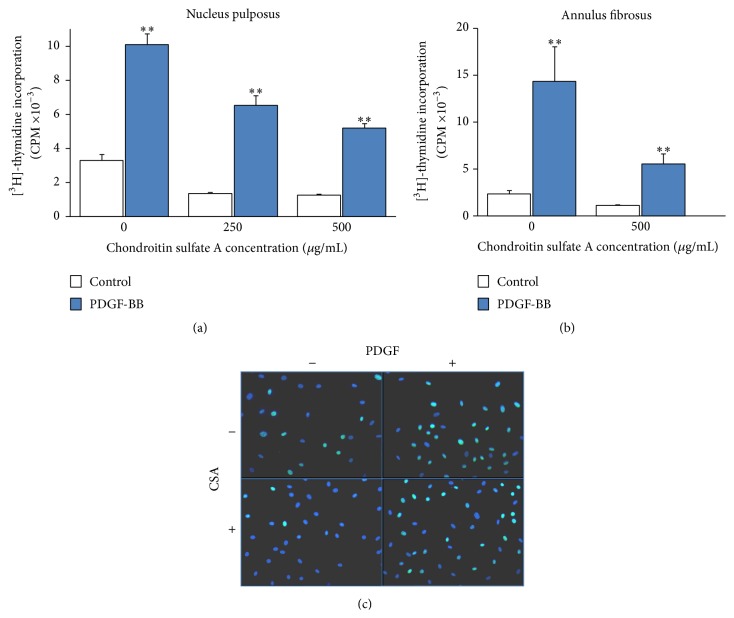
Regulation of PDGF-stimulated DNA synthesis by CSA. Bovine NP (a) and AF (b) cells were cultured in collagen gels in the presence of the indicated concentrations of CSA and stimulated with PDGF (10 ng/mL) along with tritiated thymidine for 24 hours. Thymidine incorporation was assessed as described in Section 2. Values represent mean ± SEM of three independent experiments. Asterisks (*∗∗*) indicate statistically significant differences compared to control (*p* < 0.01). In (c) monolayer cultures of bovine NP cells were stimulated with PDGF (10 ng/mL) in the presence or absence of 250 *μ*g/mL CSA along with BrdU; incorporation of the latter was assessed as described in Section 2. Images were captured from representative fields with the FITC-filter and superimposed on images captured with the DAPI-filter.

The presence of CSA in the 3D culture system was observed to inhibit dose-dependently the basal (control) tritiated thymidine incorporation (*p* < 0.01 for both concentrations); this, however, positively affected NP cell proliferative response to PDGF as percentage of the control value (Figure 2(a)). More specifically, in the plain collagen gel (i.e., in the absence of CSA) PDGF-stimulated DNA synthesis was 308%  ±20 compared to the control (*p* < 0.01), while in the presence of 250 and 500 *μ*g/mL CSA PDGF stimulation was 458%  ±41 and 412%  ±24, respectively (*p* < 0.01 for both concentrations). There was no statistically significant difference between the two CSA concentrations used (*p* = 0.09); hence the concentration of 250 *μ*g/mL was used in further experiments. Although the presence of CSA has physiological relevance only for NP, a similar experiment was performed with AF cells, and again CSA was found to suppress basal DNA synthesis without affecting PDGF stimulation (Figure 2(b)). Furthermore, a similar phenomenon was demonstrated in monolayer NP cell cultures using an alternative technique for assessing DNA synthesis, that is, BrdU incorporation (Figure 2(c) and Table 1).

### 3.2. Induction of Intracellular Signaling Pathways by Growth Factors

**Figure 3:**
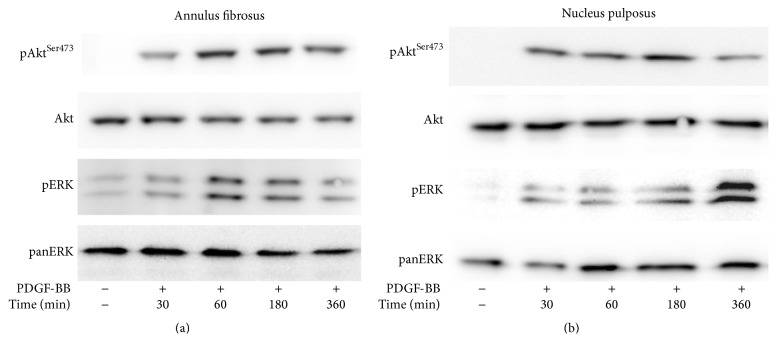
Activation of signaling pathways by PDGF. Bovine AF (a) and NP (b) cells were cultured in collagen gels and collagen gels supplemented with CSA, respectively, and stimulated with PDGF (10 ng/mL) for the indicated time intervals. Cell lysates were collected and subjected to SDS-PAGE and Western analysis as described in Section 2. One out of two independent experiments is depicted.

**Figure 4:**
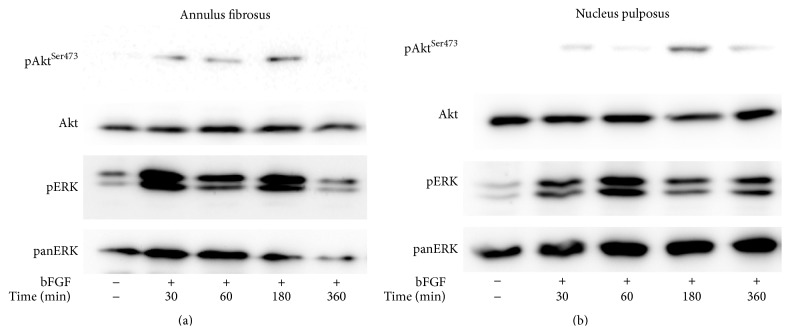
Activation of signaling pathways by bFGF. Bovine AF (a) and NP (b) cells were cultured in collagen gels and collagen gels supplemented with CSA, respectively, and stimulated with bFGF (5 ng/mL) for the indicated time intervals. Cell lysates were collected and subjected to SDS-PAGE and Western analysis as described in Section 2. One out of two independent experiments is depicted.

**Figure 5:**
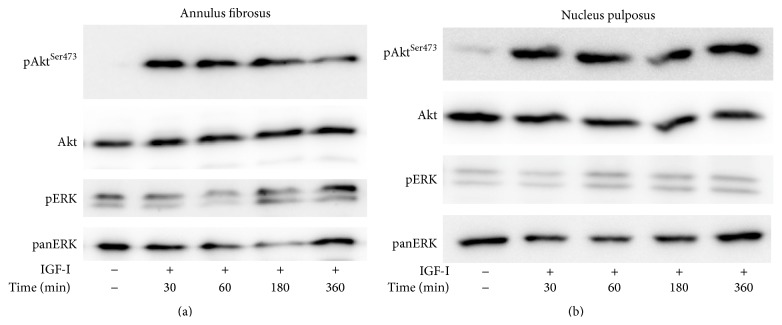
Activation of signaling pathways by IGF-I. Bovine AF (a) and NP (b) cells were cultured in collagen gels and collagen gels supplemented with CSA, respectively, and stimulated with IGF-I (100 ng/mL) for the indicated time intervals. Cell lysates were collected and subjected to SDS-PAGE and Western analysis as described in Section 2. One out of two independent experiments is depicted.

In conventional monolayer cultures, two pivotal signaling pathways have been found to mediate growth factor stimulation, that is, MEK/ERK and PI 3-K/Akt [15]. Here we report that, in 3D cultures of AF cells inside collagen gels, as well as in those of NP cells inside collagen gels supplemented with CSA, PDGF was found to phosphorylate both ERK and Akt (Figures 3(a) and 3(b)). Phosphorylation was induced rapidly and peaked at 1–3 hours, with the exception of pERK in AF cells, which peaked at 6 hours. bFGF induced immediately and intensely ERK phosphorylation, while its effect on Akt phosphorylation was less intense and it peaked at 3 hours (Figures 4(a) and 4(b)). On the other hand, IGF-I induced a less intense ERK phosphorylation peaking at 3–6 hours in the case of AF cells and at 1–3 hours in that of NP ones (Figure 5(a)). Finally, IGF-I-induced Akt phosphorylation was intense and sustained in both cell types (Figure 5(b)).

### 3.3. Response to Growth Factors in Relaxed Collagen Gels

**Figure 6:**
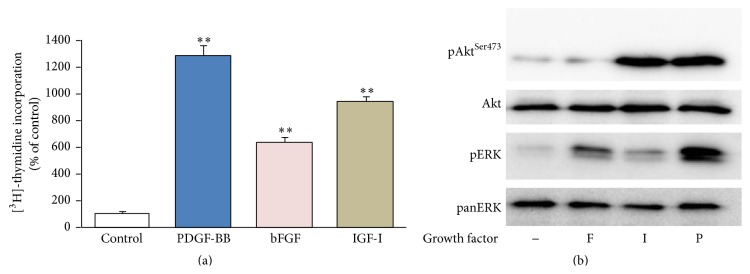
Effect of growth factors on AF cells cultured in relaxed collagen gels. Bovine AF cells were cultured in relaxed collagen gels and stimulated with 10 ng/mL PDGF-BB (P), 5 ng/mL bFGF (F), and 100 ng/mL IGF-I (I). In (a) tritiated thymidine incorporation was determined after 24 hours, while in (b) cell lysates were collected after 1 hour and subjected to SDS-PAGE and Western analysis. In (a) mean values (±SEM) of three independent experiments are shown (^*∗∗*^ *p* < 0.01), while in (b) one out of two similar experiments is presented.

The above data (Figures 1–5) were based in 3D collagen gels attached to the bottom and the wall of the culture plates, that is, stressed gels. Since there are reports from other cell types that relaxation of the collagen gel may affect the response to a growth factor [20], we have tested the response of AF cells cultured in floating collagen gels—detached from the bottom and the wall of the culture plate—to the three growth factors under study. As shown in Figure 6(a), AF cells in relaxed gels exhibit qualitatively similar proliferative responses to all three growth factors compared to cells in stressed gels (Figure 1(a)). Although stimulation in relaxed gels was higher than that in stressed gels for each growth factor, this difference was statistically significant only in the case of bFGF; more specifically the values were 1282%  ±82 versus 746%  ±150 (*p* = 0.058) for PDGF, 640%  ±37 versus 301%  ±66 (*p* = 0.013) for bFGF, and 942%  ±37 versus 620%  ±125 (*p* = 0.144) for IGF-I. As shown in Figure 6(b), in AF cells cultured inside relaxed collagen gels the ERK and Akt signaling pathways were activated by the three growth factors in an extent similar to that observed in the case of stressed gels (compare to Figures 3(a), 4(a), and 5(a)).

### 3.4. Contribution of Various Signaling Pathways to the Proliferative Response Elicited by Growth Factors

**Figure 7:**
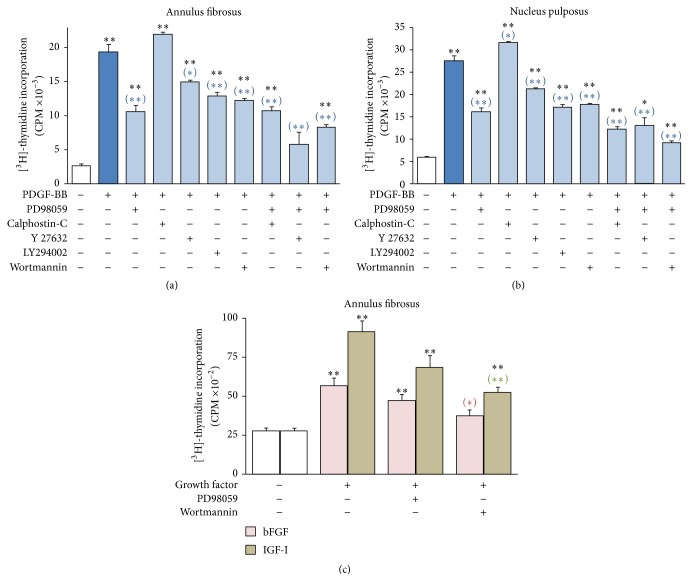
Inhibition of growth factor-induced DNA synthesis through blocking of signaling pathways. Bovine AF ((a) and (c)) and NP (b) cells cultured in collagen gels and collagen gels supplemented with CSA, respectively, were preincubated with PD98059 (25 *μ*M), calphostin-C (100 nM), Y 27632 (10 *μ*M), LY294002 (10 *μ*M), wortmannin (100 nM), or the indicated combinations of them for 45 minutes. They were then stimulated with the indicated growth factors, and tritiated thymidine incorporation was determined after 24 hours, as described in Section 2. Mean values (±SEM) of three independent experiments are shown. Black asterisks indicate statistically significant differences compared to control, while blue, red, and green asterisks in parentheses indicate statistically significant differences compared to PDGF-BB, bFGF, and IGF-I, respectively (^*∗∗*^ *p* < 0.01; ^*∗*^ *p* < 0.05).

As shown above, the ERK and Akt pathways are activated by PDGF, bFGF, and IGF-I in AF and NP cells in 3D cultures. To determine whether these signaling pathways mediate the proliferative responses elicited by these three growth factors, DNA synthesis was monitored in the presence of the pharmacologic inhibitors PD98059 and wortmannin, blocking MEK/ERK and PI 3-K/Akt, respectively. The PI 3-K inhibitor LY294002 was also used for verification. Furthermore, the inhibitors calphostin-C and Y 27632, blocking Protein Kinase-C (PKC) and Rho-Associated Protein Kinase (ROCK), respectively, were also employed, since both signaling entities have been implicated in the maintenance of chondrocyte differentiation in 3D cultures [21, 22]. Since PDGF was found to be the most potent among the three growth factors (Figure 1), furthermore activating intensely both ERK and Akt (Figure 3), we studied the effect of the various signaling inhibitors on PDGF-induced DNA synthesis. In both AF and NP 3D cell cultures, PD98059 was the most potent inhibitor, followed by wortmannin and LY294002 (Figures 7(a) and 7(b)). Y 27632 was less potent, while calphostin-C had no effect at all. None of the inhibitors was blocking totally DNA synthesis induced by PDGF. Since PD98059 was the most potent among the inhibitors, we studied also its combinations with other inhibitors and observed that Y 27632 statistically significantly enhanced its inhibitory effect in AF cells (Figure 7(a)), and wortmannin did so in NP cells (Figure 7(b)). Collectively, these results indicate that, among the signaling pathways examined, the most important ones for mediating the growth stimulatory activity of PDGF are MEK/ERK and PI 3-K/Akt. These pathways were found to mediate also the proliferative effects of bFGF and IGF-I, both in AF cells (Figure 7(c)) and NP ones (not shown). The main difference with the case of PDGF was that wortmannin exerted a more potent inhibitory effect compared to PD98059 on the growth stimulation induced by bFGF and IGF-I.

## 4. Discussion

The aim of this study was to examine the growth response of IVD cells cultured in 3D culture systems—simulating the in vivo environment of the tissue—to PDGF, bFGF, and IGF-I. This effort was triggered by various observations in the literature indicating that IVD cells in monolayer culture have the tendency to dedifferentiate [23] similarly to chondrocytes [24]. Hence, many studies have been conducted in the very popular system of alginate beads, that is, a negatively charged gelatinous substance forming a 3D matrix [25] and resembling the negatively charged (due to the proteoglycans) milieu of the NP. On the other hand, this environment is far from the original extracellular matrix of the disc and most importantly is not very suitable for studying the proliferative response to growth factors, since IVD cells in alginate only marginally proliferate in response to serum [26]. Accordingly, collagen type-I gels offer a 3D environment containing one of the most abundant ECM constituents of the disc—at least regarding AF [4]—and they have been used extensively for the culture of chondrocytes, especially for tissue engineering purposes [27, 28]. Notably, 3D collagen gels have been shown to permit the proliferation of chondrocytes, although not at the rate observed in monolayer cultures [29]. Furthermore, in order to better simulate the NP environment, we have added in the collagen solution the glycosaminoglycan chondroitin sulfate (CSA), an abundant constituent of NP proteoglycans [30].

The data presented here show that in the 3D environments we have employed both AF and NP cells exhibit an intense proliferative response to PDGF, bFGF, and IGF-I (Figure 1). Comparing these results with our previous study in monolayer cultures [15], one can see that the proliferative responses in the 3D environments are more intense than those in monolayers—as percentages of the respective control values—with the exception of PDGF acting on NP cells. This may be the result of the lower basal DNA synthesis levels observed in the control 3D cultures, at least regarding AF cells, in agreement with previous observations in other cell types concerning the growth restraining properties of the polymerized collagen [19, 31]. On the other hand, the more intense response to growth factors in the 3D environments may simply reflect an increased complexity of the network of activated signaling molecules compared to the monolayer cultures [32]. In general, the intensity of the proliferative responses to each growth factor in the 3D culture environments follows a similar pattern with that in monolayers, that is, PDGF > IGF-I > bFGF (Figure 1; [15, 16]).

The supplementation of collagen gels with CSA led to a further inhibition of basal DNA synthesis levels (Figures 2(a) and 2(b)), a fact observed also in monolayer cultures (Figure 2(c) and Table 1), in agreement with reports in the literature coming from different cell types [33, 34]. Furthermore, CSA has been shown to inhibit PDGF-induced proliferation of human lung fibroblasts [35] while stimulating that of human fibrosarcoma cells [36]. Nevertheless, in the present study and in a 3D culture environment, the proliferative responses of NP cells to PDGF (Figure 2) and to bFGF and IGF-I (not shown here) were maintained; actually they were higher in terms of percentages of the control values (see Section 3; Section 3.1). In the same direction, a growth promoting synergistic effect of chondroitin sulfate was observed for chondrocytes cultured in polyvinyl alcohol hydrogels and stimulated to proliferate with serum [37].

All three growth factors studied were found to induce two signaling entities of major importance regarding the regulation of cell proliferation, that is, ERK and Akt (Figures 3–5). Once again the patterns of ERK and Akt activation resembled those observed previously in monolayer cultures [15, 16], such as the intense activation of both pathways by PDGF, the less intense phosphorylation of ERK by IGF-I, and the much less intense activation of Akt by bFGF (Figures 3–5; see also Figure 6(a)). To our knowledge, this is the first study presenting data on the activation of signaling pathways in 3D cultures of IVD cells in response to these three growth factors. Phosphorylation of ERK and Akt in response to IGF-I has been shown previously in human articular chondrocytes both in monolayer cultures and in alginate beads [38].

Since mechanical stressing represents an integral part of IVD homeostasis [39], in an effort to identify any possible interference of the mechanical tensile forces with IVD cell responses to growth factors, we have tested cultures in relaxed collagen gels in addition to stressed ones. Interestingly, the responses of IVD cells to PDGF, bFGF, and IGF-I were found to be qualitatively similar both in stressed and in relaxed 3D collagen gels, in terms of DNA synthesis as well as intracellular signaling pathway activation (Figure 6). Our data may be at variance with reports from human skin fibroblast cultures, where the response to PDGF changes dramatically from stressed to relaxed collagen gels [20]; however in that case the two different culture environments correspond to two diverse phases of skin repair, that is, the granulation tissue versus the reconstituted dermis [40].

In our previous study conducted in monolayer cultures, we have shown that blocking the MEK/ERK or the PI 3-K/Akt signaling pathways results in a considerable inhibition of the growth stimulatory effects of PDGF, bFGF, and IGF-I on IVD cells, while the simultaneous inhibition of the two pathways completely blocks DNA synthesis induction by the three growth factors [15]. Our present observations from 3D cultures suggest that these two pathways are indeed very important for mediating the growth stimulatory effects of the three growth factors (Figure 7); however even the simultaneous blockade of MEK/ERK and PI 3-K/Akt did not completely inhibit growth factor-induced DNA synthesis, suggesting the involvement of more signaling molecules. The latter, however, does not belong to PKC, since its inhibition did not contribute more to the inhibitory effects of the MEK/ERK inhibitor PD98059 (Figure 7). ROCK, on the other hand, seems to be implicated only in the case of AF cells (Figure 7(a)), a fact possibly related to the intense tensile forces these cells are experiencing. Our future plans include the study of possible candidates for interfering with growth factor signaling in the IVD, such as several members of the integrin family, as well as focal adhesion kinase [41–44].

In conclusion, the present study indicates that IVD cells cultured in 3D organotypic gels respond to PDGF, bFGF, and IGF-I in terms of DNA synthesis stimulation, through the involvement of the pivotal MEK/ERK and PI 3-K/Akt signaling pathways. The proposed culture systems of collagen type-I gels for AF cells and CSA supplemented collagen gels for NP cells may prove useful for the in vitro proliferation and/or the delivery of cells aiming at the design of novel therapies for the regeneration of the degenerated IVD.

## Figures and Tables

**Table 1:** Regulation of PDGF-stimulated DNA synthesis in monolayer NP cultures by CSA^a^.

CSA concentration (*μ*g/mL)	PDGF-BB concentration (ng/mL)	
	0	10
0	34.5% (±2.5)	58.1% (±2.1)
250	10.2% (±3.6)	46.2% (±10.2)

^a^Percentage of BrdU-positive nuclei (mean ± SEM from three independent cultures; three representative fields were counted in each culture).
